# A Novel Terahertz Metamaterial Microfluidic Sensing Chip for Ultra-Sensitive Detection

**DOI:** 10.3390/nano14131150

**Published:** 2024-07-04

**Authors:** Yuan Zhang, Keke Jia, Hongyi Ge, Xiaodi Ji, Yuying Jiang, Yuwei Bu, Yujie Zhang, Qingcheng Sun

**Affiliations:** 1 Key Laboratory of Grain Information Processing and Control, Ministry of Education, Henan University of Technology, Zhengzhou 450001, China; zhangyuan@haut.edu.cn (Y.Z.);; 2 Henan Provincial Key Laboratory of Grain Photoelectric Detection and Control, Zhengzhou 450001, China; 3 College of Information Science and Engineering, Henan University of Technology, Zhengzhou 450001, China; 4 School of Artificial Intelligence and Big Data, Henan University of Technology, Zhengzhou 450001, China

**Keywords:** terahertz, metamaterials, microfluidic sensors, ultrasensitive detection

## Abstract

A terahertz metamaterial microfluidic sensing chip for ultrasensitive detection is proposed to investigate the response of substances to terahertz radiation in liquid environments and enhance the molecular fingerprinting of trace substances. The structure consists of a cover layer, a metal microstructure, a microfluidic channel, a metal reflective layer, and a buffer layer from top to bottom, respectively. The simulation results show that there are three obvious resonance absorption peaks in the range of 1.5–3.0 THz and the absorption intensities are all above 90%. Among them, the absorption intensity at M1 = 1.971 THz is 99.99%, which is close to the perfect absorption, and its refractive index sensitivity and Q-factor are 859 GHz/RIU and 23, respectively, showing excellent sensing characteristics. In addition, impedance matching and equivalent circuit theory are introduced in this paper to further analyze the physical mechanism of the sensor. Finally, we perform numerical simulations using refractive index data of normal and cancer cells, and the results show that the sensor can distinguish different types of cells well. The chip can reduce the sample pretreatment time as well as enhance the interaction between terahertz waves and matter, which can be used for early disease screening and food quality and safety detection in the future.

## 1. Introduction

Terahertz (THz) waves refer to electromagnetic waves with frequencies 0.1–10 THz and lie between microwave and infrared bands of the electromagnetic spectrum [1,2]. The energy of THz waves is one million times smaller than that of X-rays, which means that they do not cause radiation damage upon penetrating substances [3]. In addition, the vibrational and rotational energy levels of many semiconductors, plasmas, and biomolecules fall in the THz band range with unique spectral fingerprints [4]. In recent years, terahertz time-domain spectroscopy (THz-TDS) has become an effective means of detecting substances. THz-TDS has been used in medical diagnosis and the qualitative and quantitative analysis of agricultural products, cultural relics, and drugs [5,6,7]. However, the interaction between THz waves and substances is weak due to the lack of efficient terahertz sources and sensitive detectors. This limitation is currently a bottleneck in detecting micro or trace elements.

Metamaterials (MMs) are composite structures or materials consisting of periodic or non-periodic artificial atomic arrangements with optical and mechanical properties not found in natural materials, such as super-lens [8], negative refractive indices [9], and negative electromagnetic response [10], which has led to new fields being explored in electromagnetic studies, ranging across the entire frequency spectrum from zero to the far optical [11]. In recent decades, metamaterial structures have been widely used in the fields of sensing, spectroscopy, medicine, and imaging [12]. The metamaterial-based terahertz sensors can enhance the local electromagnetic field and, eventually, the interaction between terahertz waves and matter. Moreover, terahertz sensors based on MMs are label-free and affinity sensors, providing new ways of detecting trace samples [13,14,15]. Currently, the focus is drawn to filter-type and absorber-type sensors, improving the performance of sensors and designing multi-band sensors to study the detection mechanism of substances [16,17,18,19]. Nevertheless, the strong absorption of terahertz waves by polar solutions such as water remains unresolved. Drying the sample before taking the measurement to eliminate the influence of liquids is useless because the sample’s structure can change during the drying process. Therefore, obtaining THz-related measurements in the actual environment of biological samples is challenging.

A microfluidic chip confines a nanoliter to a microliter of microfluid in a single chamber through microfluidic channels. Confining a small quantity of fluid reduces the amount of sample required for testing, improves the reaction efficiency, and enhances interaction with the target biomolecules in the aqueous environment [20,21,22]. In this study, a terahertz metamaterial microfluidic sensing chip (TMMSC) for liquid sample detection is designed and analyzed. We use numerical simulations to explore the sensing mechanism of TMMSC. Moreover, the electromagnetic characteristics of the sensor in the terahertz band are explored. The stability and detection of the sensor are also evaluated. It is shown that the sensor designed in this study possesses excellent detection capability, wide-angle insensitivity, and free space operation. Additionally, the proposed sensor discriminates cancer cells from healthy cells very well. TMMSC needs a small amount of sample for testing and offers high sensitivity, ultra-miniaturization, no radiation damage, and deep interaction with the sample. The developed sensor is relevant in early disease screening and food quality testing applications.

## 2. Structural Design and Simulation

**Figure 1:**
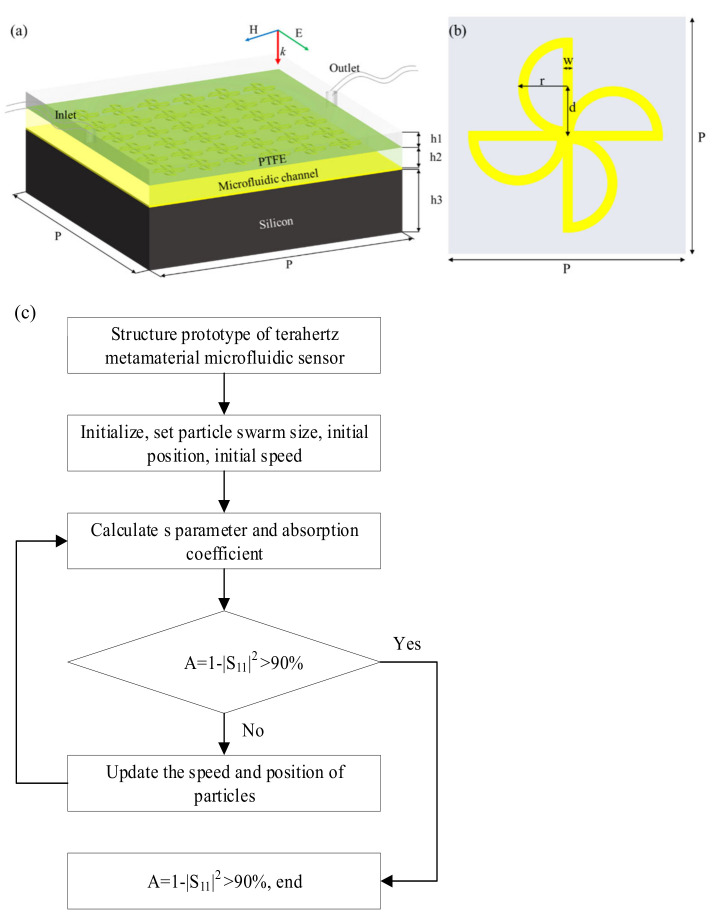
(**a**) TMMSC under TE-polarized terahertz wave irradiation. (**b**) Metallic microstructure. (**c**) Structural design diagram of microfluidic sensor based on the particle swarm optimization algorithm.

A simple structure with a multi-band resonator was obtained based on a cross-type structure. The overall structure of TMMSC designed in this study is shown in Figure 1a. The unit structure has five components: a cover layer, a metallic microstructure, a micro-flow channel, a metallic reflective layer, and a buffer layer. Figure 1b shows a magnified view of the metallic microstructure. The cover layer material is polytetrafluoroethylene (PTFE) with a dielectric constant of 2.1 + i0.0002 [23]. The metallic microstructure and the metallic reflective layer are made of gold with a conductivity of 4.561 × 10^7^ S/m [24]. The buffer layer is made of high-resistance silicon as a substrate with a dielectric constant of 11.9 + i0.00025 [24,25].

We used CST MWS 2020 for the numerical simulation of the structure. In the simulation setup, a frequency domain solver based on the finite element algorithm was used to solve Maxwell’s differential equations and divide the mesh with a tetrahedral network [26]. In the boundary setting, the x-y plane was set as the periodic boundary, and the z-direction was set as the open boundary to simulate an infinitely large periodic array. Terahertz waves are incident vertically on the surface of the TMMSC. The particle swarm optimization (PSO) algorithm is a kind of swarm intelligence algorithm, which evaluates particles through fitness function, updates the speed and position of particles, and finds the optimal solution. In the range of 1.5~3.0 THz, the aim of this paper is to find the matrix corresponding to the highest peak of the absorption peak with an absorption rate greater than 90%. The PSO is used to optimize the seven parameters of the microfluidic sensor in the CST-MATLAB co-simulation to obtain the ideal absorption. The proposed particle swarm optimization algorithm has stronger adaptability. It can quickly optimize the parameters of the windmill-type microfluidic sensor, such as cell structure period, thickness, linewidth and so on, and reduce the number of iterations, so as to more accurately and quickly design the terahertz metamaterial microfluidic sensor with ideal absorption characteristics. The optimal design of terahertz metamaterial microfluidic sensor based on the particle swarm optimization algorithm is shown in Figure 1c. The optimized structural parameters are shown in Table 1.

Three metrics are usually used to evaluate sensor performance: Q-factor, sensitivity, and figure of merit (FOM) [27]. The Q-factor is an important index for evaluating the sensor. The following formula calculates the Q-factor. Q = ƒ/FWHM. In this formula, ƒ indicates the resonant frequency and FWHM is the full width at half maximum. The higher the Q-factor, the higher the spectral resolution of the sensor and the better the performance. Currently, many researchers are focusing on enhancing the Q-factor of sensors as a research objective [28,29,30,31]. Sensitivity is a crucial indicator of sensor performance. It is calculated from the formula ∆ƒ/∆n, where ∆ƒ represents the shift in the frequency response of the substance relative to the null sensor and ∆n is the change in the refractive index of the substance relative to that in air. The sensitivity indicates the shift in the sensor frequency caused by a unit refractive index substance in GHz/RIU. A higher sensitivity means a better ability to detect the substance. FOM value is a comprehensive evaluation index calculated by S/FWHM. An enormous FOM value represents a better overall performance of the sensor.

**Figure 2:**
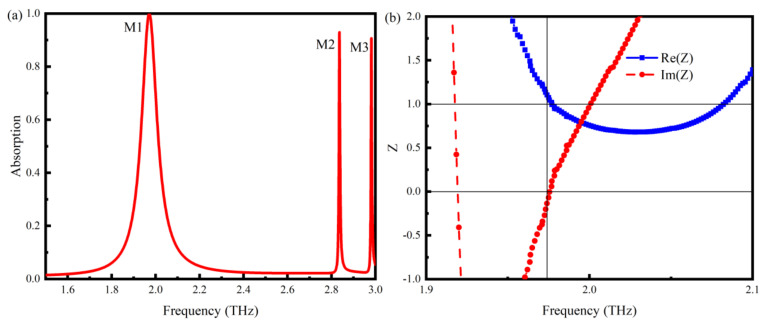

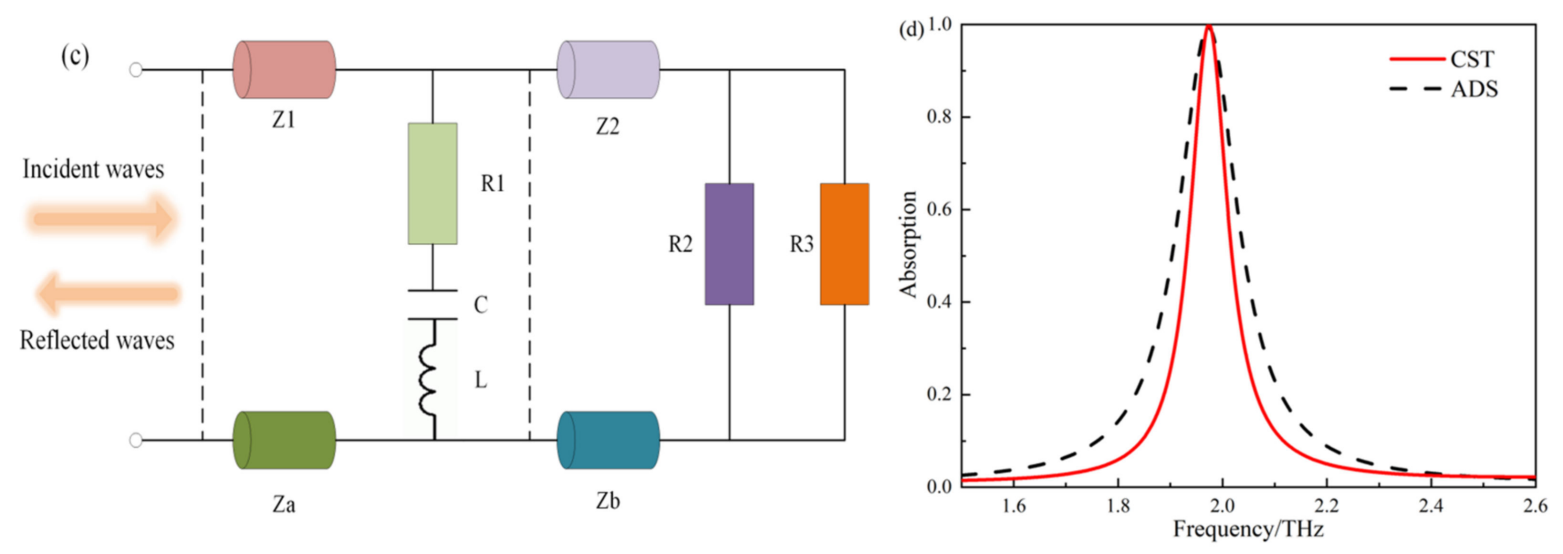
(**a**) TMMSC characteristic absorption curve. (**b**) the relative impedance of TMMSC in M1. (**c**) RLC equivalent circuit model. (**d**) Simulated absorption from ADS and CST.

The S-parameters are obtained by numerical simulation, where *S*_11_ denotes the reflection coefficient and *S*_21_ denotes the transmission coefficient. Since the metal reflector layer reflects the THz wave completely, the TMMSC transmittance $T\left(\omega \right)={\left|S_{21}\right|}^{2}=0$. The absorption is calculated by $A\left(\omega \right)=1-R\left(\omega \right)-T\left(\omega \right)=1-{\left|S_{11}\right|}^{2}$. The characteristic absorption curve without a sample in the microfluidic channel is shown in Figure 2a. In the frequency range of 1.5–3.0 THz, there are three obvious resonance absorption peaks of TMMSC, M1 = 1.971 THz, M2 = 2.836 THz, and M3 = 2.981 THz. The overall absorption of TMMSC is above 90%. However, the absorption of the M1 peak is more than 99.99%. According to the equivalent model theory [32], the relative impedance of the TMMSC can be calculated by the S parameter as $Z=\sqrt{\frac{{\left(1+S_{11}\right)}^{2}-S_{21}^{2}}{{\left(1-S_{11}\right)}^{2}-S_{21}^{2}}}$. It can be simplified to $Z=\left(1+S_{11}\right)/\left(1-S_{11}\right)$, since *S*_21_ = 0. The relative impedance of the M1 mode is shown in Figure 2b, where the real part approaches one, and the imaginary part approaches zero. The impedance matching with free space is achieved. According to the equivalent circuit model, the proposed cell array can generally be regarded as a static series RLC circuit. The equivalent capacitance of the array is mainly a result of the strong electric field distribution inside the metamaterial; the equivalent inductance is mainly a result of the semicircular metal ring; the equivalent resistance is mainly a result of the the dielectric loss and ohmic loss of the material; and the different dielectric layers in the middle can be regarded as having the characteristic impedance Zn. The equivalent circuit is shown in Figure 2c.

Using ads circuit simulation software, the absorption curve of the microfluidic sensor is calculated, as shown in Figure 2d. Comparing the ads circuit simulation results with the CST full wave simulation results, the absorption curve is relatively consistent, and there is only a difference in the bandwidth. The main reasons are that the calculation process from lumped parameters to geometric parameters is ignored, and the accuracy and algorithm of the circuit simulation software and full wave simulation software are different.

## 3. Analysis of the Numerical Results

### 3.1. Physical Mechanisms

**Figure 3:**
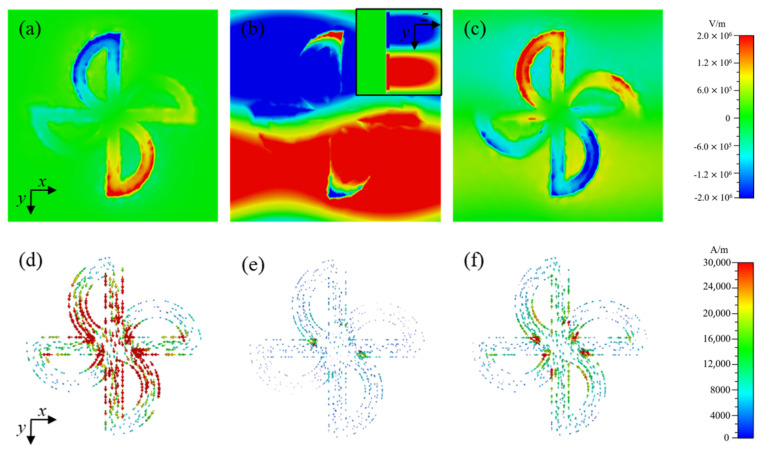
Electric field along the z-plane and surface current distribution for a sample-free resonance in the TMMSC microfluidic channel. (**a**) Re(Ez) of M1, (**b**) Re(Ez) of M2, (**c**) Re(Ez) of M3, (**d**) surface current of M1, (**e**) surface current of M2, and (**f**) surface current of M3 resonance peaks.

Let us explain the three resonance peaks of TMMSC in the range of 1.5–3.0 THz. Figure 3 shows the electric field and the surface current distribution at the resonance frequencies of the sensor using the metallic microstructure of the windmill model. From Figure 3a,d, it can be seen that at 1.971 THz, the electric field is mainly distributed in the microstructure, and the intensity of the surface current is higher along the vertical arm of the windmill structure because the direction of the applied electric field is parallel to the y-axis. In addition, the electric field intensity is shown in the form of “+” and “−” signs. This means that M1 is a typical dipole resonance. Figure 3b shows that the electric field is mainly distributed in the microstructure with PTFE as the cover material. The box plot indicates the electric field distribution in the y-z plane. Moreover, Figure 3e shows that the current is minimal. Therefore, the M2 resonance may be caused by internal losses in the dielectric material. From Figure 3c,f, it can be seen that the electric field is primarily distributed in the vertical semicircle of the microstructure with a small value of current intensity. In addition, there is a partial electric field in the cover material. Therefore, destructive interference between unit structures or internal material loss is the leading cause of M3 resonance.

### 3.2. Stability Analysis

**Figure 4:**
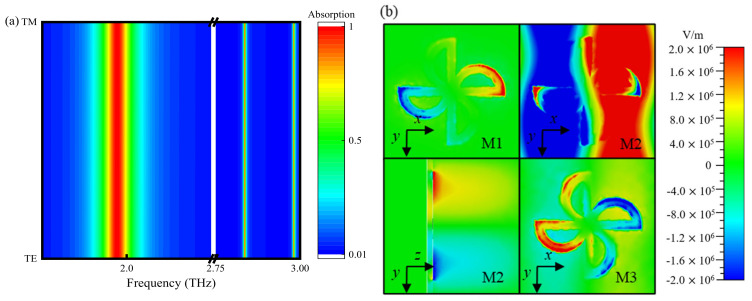
(**a**) Absorption characteristics of TE and TM polarized THz waves. (**b**) TM polarized electric field distribution.

The polarization angle and incident angle dependency of the sensor are essential indicators of the stability of the sensor. The electromagnetic response curves of TE and TM polarized THz waves incident perpendicular to the TMMSC surface are shown in Figure 4a. Due to the centrosymmetric pattern of the unit structure, the TMMSC exhibits excellent performance for the two types of incident polarization waves. In addition, we also analyzed the electric field distribution of the TMMSC in TM mode, as shown in Figure 4b. It is observed that the electric field distribution changes with the direction of polarization compared to the electric field distribution of the THz wave incident perpendicular to the sensor surface under TE polarization.

**Figure 5:**
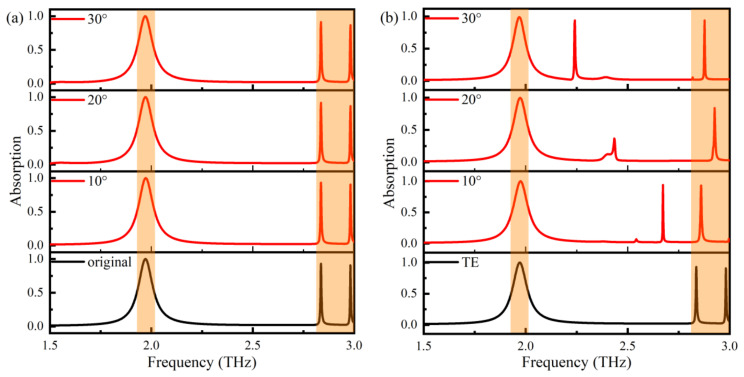
(**a**) Variation of absorption with phi for theta = 0°. (**b**) Variation of absorption with theta for phi = 0°.

Figure 5 shows the absorption curves when the polarization and incident angles vary in TE polarization modes. From Figure 5a, it is evident that the absorption curve changes negligibly in the 30° polarization range. The TMMSC is polarization insensitive in the 30° polarization range. Moreover, it also shows that the sensor performance for detection will remain consistent even if the sensor is angularly displaced due to improper operation. When theta varies from 0°–30° for phi = 0°, the incident THz wave changes from normal to oblique incidence with respect to the TMMSC surface. The absorption curve is shown in Figure 5b. The oblique incidence angle has a more substantial influence on TMMSC. The resonance frequency and absorption of the M1 peak are more stable than the other two peaks for a wide incidence angle range. The resonance peak M2 shows variable characteristics. Its resonance frequency moves to lower frequencies with the increase in the incident angle, and the absorption variation is irregular. The resonance peak M3 has a more stable absorption intensity, and the resonance frequency moves within a specific range. According to the electromagnetic response of the three resonance peaks with the change in the polarization angle and incidence angle, resonance peak M1 has polarization and incidence angle insensitive characteristics and can be the best mode for sensing the signal detection.

### 3.3. Effect of Structural Parameters

The height of the microfluidic channel is an important parameter that cannot be neglected in the sensor design process. Once the transverse dimensions of the sensor have been determined, the height of the microfluidic channel determines the performance of the sensor and the sample feed volume. This section discusses the effect of microfluidic channel height on the electromagnetic response of the sensor to determine the optimal channel height.

**Figure 6:**
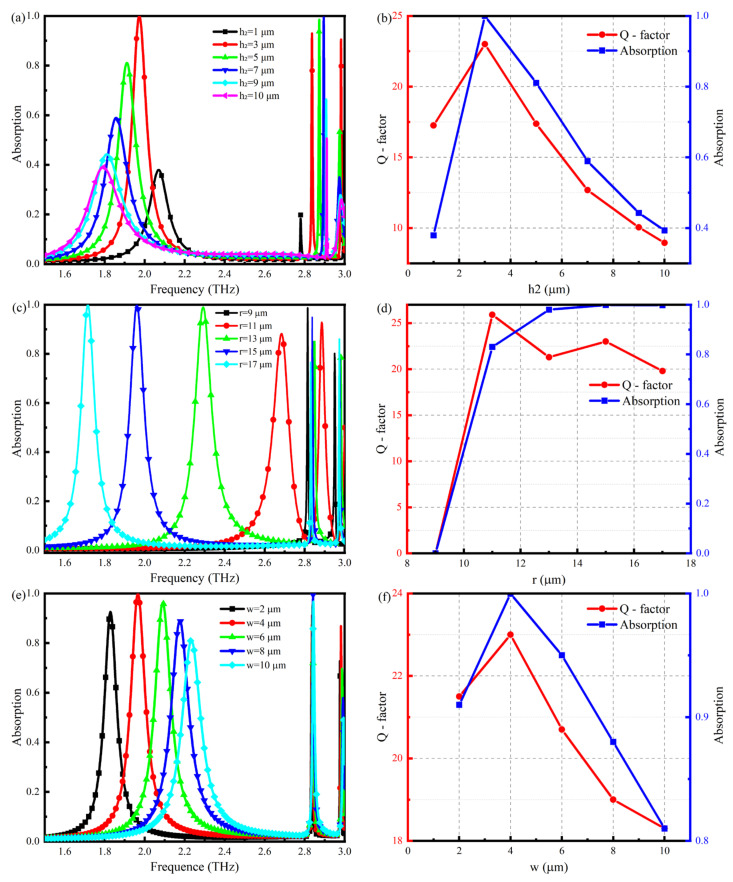
(**a**) TMMS absorption characteristics for different h2; (**b**) variation of Q-factor and absorption intensity of M1 with h2. (**c**) TMMS absorption characteristics for different r; (**d**) variation of Q-factor and absorption intensity of M1 with r. (**e**) TMMS absorption characteristics for different w; (**f**) variation of Q-factor and absorption intensity of M1 with w.

Figure 6a shows the absorption spectra of TMMS when h2 is increased from 1 μm to 10 μm. It can be seen from the figure that the M1 resonance peak changes significantly with the increase in h2. First, the resonance frequency shifts to a low frequency. Second, the absorption intensity first increases and then decreases. Similarly, the resonance peak M2 moves to high frequencies when h2 increases from 1 μm to 10 μm, but not substantially. The absorption intensity increases and then decreases. For resonance peak M3, only a slight change in the resonance frequency of 20 GHz is observed. However, there is a significant change in absorption intensity of M3 when h2 increases from 1 μm to 10 μm, reaching the absorption maximum at 3 μm. Figure 6b shows the variation in Q-factor and absorption with respect to h2 for the M1 resonance peak. The highest Q-factor and absorption can be seen when h2 = 3 μm. The magnitude of the Q-factor determines the sensor’s sensitivity to a considerable extent. Therefore, it is concluded that the sensor will have the best detection capability when the height of the microfluidic channel is 3 μm. For a TMMS of 1 cm × 1 cm, the injection volume is only 3 × 10^−7^ L, which enables the detection of micro or trace samples in a minute quantity.

Moreover, in order to better understand the performances of the sensor structure, the geometric sizes (r,w) of the TMMS structure are researched and illustrated in Figure 6. Figure 6c shows the effect of the radius r of the metal microstructure on the sensor. When r increases from 9 μm to 17 μm, the resonant frequency of M1 mode moves to the lower frequency, and a redshift phenomenon occurs. When r = 9 μm, M1 mode does not exist, and the absorption intensity of M1 mode gradually increases with the increase in r. Figure 6d shows the changes in the Q-factor and absorption intensity of M1 with r. When r = 15 μm, the absorption intensity reaches the maximum, and the absorption intensity tends to stabilize as r continues to increase, while the Q-factor begins to decrease. Therefore, the choice of r = 15 μm. The influence of the linear width w of the metal microstructure on the sensor is shown in Figure 6e. It can be seen that with the increase in w, the resonant frequency of mode M1 moves towards high frequency and blue shift occurs, and the absorption intensity increases first and then decreases. The resonant frequency of mode M2 does not change obviously, moves to high frequency, and the absorption intensity increases gradually. The variation law of model M3 is the same as that of model M1: the absorption intensity increases first and then decreases gradually. Figure 6f shows the variation in the Q-factor and absorption intensity of mode M1 with w. When w = 4 μm, the Q-factor and absorption intensity of M1 reach the maximum, at which time the performance of the sensor is optimal. The above results show that when the geometric parameter error of the sensor structure is within a certain range, the sensor structure can still maintain excellent sensing performance and has a certain stability.

### 3.4. Sensing Performance

The equivalent capacitance of the sensor (C_eff_) depends on the capacitance of the device. The capacitance generated for the sample (C_sensor_) and the capacitance of the device do not change when the structural dimensions of the sensor are determined [33]. Therefore, changes in the refractive index of the analyte cause changes in the surrounding dielectric environment, which alter the C_sensor_ and affect the electromagnetic response of the sensor, such as its resonance frequency and absorption. In analyzing the sensing performance of the sensor, samples with different thicknesses and dielectric constants were placed on the surface of the sensor. The detection of the samples was achieved by monitoring the shift in the resonance frequency of the sensor and the change in the absorption.

**Figure 7:**
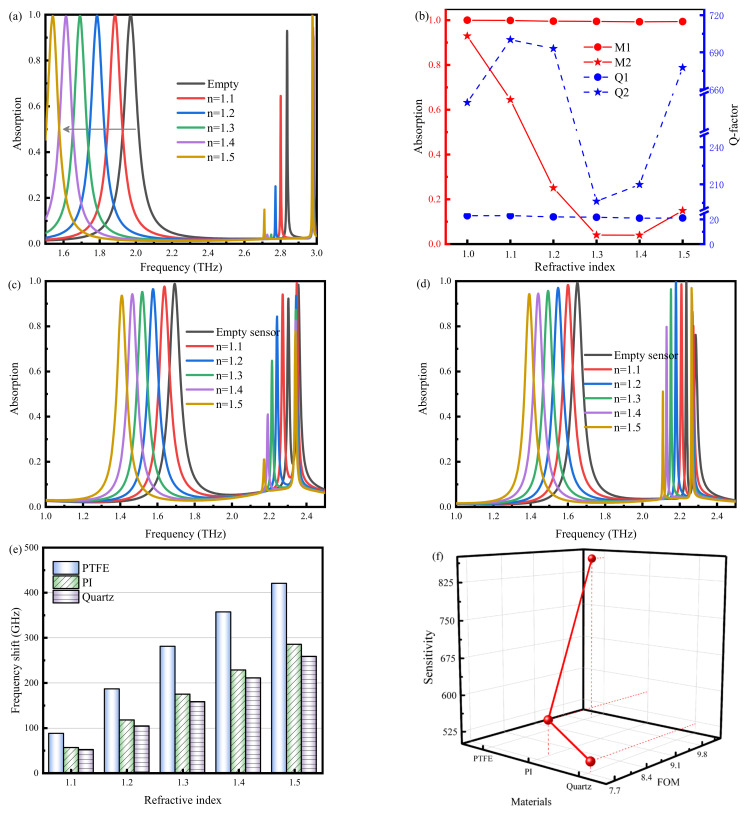
(**a**) Absorption curves with and without sample filling in the microfluidic channel. (**b**) Absorption and Q-factor of M1 and M2 resonance peaks. (**c**) The detection capability of PI-type sensor. (**d**) The detection capability of quartz-type sensor. (**e**) The frequency shift of the M1 resonance peak. (**f**) Sensitivity and FOM of M1 resonance peak.

The sensing detection capability of TMMSC was evaluated by adding analytes of different refractive indices to the microfluidic channel, as shown in Figure 7. The absorption curves of TMMSC as the refractive index of the analyte changes from 1.1 to 1.5 are plotted in Figure 7a. The absorption curve of resonance peak M1 changes the most, and the resonance redshift increases as the refractive index of the analyte increases. It is easy to understand that the M1 resonance peak is the most sensitive to the change in the refractive indices of the analyte and has better detection. In contrast, the resonance peak M3 changes negligibly with the change in the refractive indices of the analyte. The absorption intensity and Q-factor of M1 and M2 are shown in Figure 7b. Compared with the M2 resonance peak, the absorption and Q-factor of the M1 resonance peak are stable, and the absorption remains above 99% with the change in the refractive index of the substance. Therefore, the absorption is not detected as a sensing signal. Although the M2 resonance peak has a high Q-factor, the stability of its absorption is weak. Due to this, the M2 resonance peak is not helpful in a complex detection environment. The M1 resonance peak has a high detection stability.

In addition, the effect of different cover materials on the sensor was also analyzed. Figure 7c,d show the absorption curves for Polyimide (PI, ε = 3.5 + i0.0027) [34] and quartz (ε = 3.75 + i0.0004) [35] as cover materials. Similar to the PTFE-type cover layer, the frequency of the M1 resonance peak shifts significantly. Moreover, the absorption of the M2 resonance peak changes a lot. Figure 7e. plots the frequency shift of the three cover materials, PTFE, PI, and quartz. For resonance peaks M1, the frequency shift with PTFE cover material is significantly larger than that of PI and quartz. Since the frequency shift of the M2 resonance peak is similar to the frequency shift of the PTFE, it is not plotted. TMMSC was evaluated by calculating the sensitivity and FOM value of M1, as shown in Figure 7f. The results showed that the sensitivity and FOM value of PTFE were higher than that of 859 GHz/RIU and 10, respectively. The sensitivity and FOM values with PI and quartz cover materials are 571 GHz/RIU and 7.9, and 520 GHz/RIU and 7.8, respectively. These results verify that the smaller the dielectric constant of the dielectric material, the better the sensor performance. Table 2 lists the performance of the proposed sensor compared with previous sensors in the literature. The sensor proposed in this study has significant advantages regarding Q-factor, sensitivity, and FOM values. It can provide a reference for designing sensors with simple structures and superior performance.

### 3.5. Cancer Cell Sensing Simulation Experiment Based on Refractive Index

**Figure 8:**
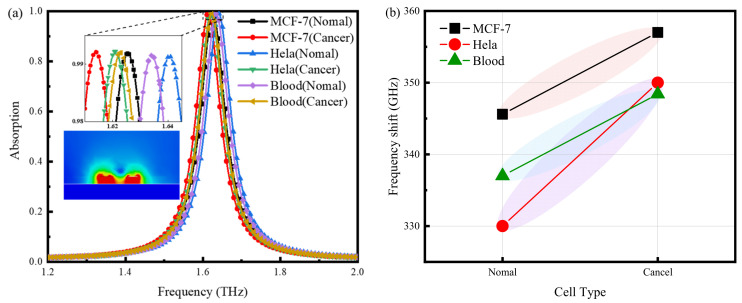
(**a**) Absorption curve of TMMSC when filling cancer and normal cells. (**b**) Frequency shift relationship curve.

Cells reveal the past, present, and future of an organism’s health and are essential for the early detection of disease [41,42]. In this section, let us evaluate the sensor by discussing the absorption characteristics of TMMSC with different types of cells in different tissues of the human body. The injection volume is only 0.3 μL because the sensor size is 1 cm × 1 cm, and the terahertz spot is about 5 mm × 5 mm. The refractive indices of different cell types are shown in Table 3. The absorption spectra of different cells are shown in Figure 8. Section 3.2 and Section 3.3 show that the M1 resonance peak is polarization- and incidence angle-insensitive, with the highest sensitivity to the analyte. Therefore, the M1 resonance peak was used to observe the sensing signal. Several types of cells can be distinguished according to their absorption curves, as shown in Figure 8a. The inset shows the electric field distribution of TMMSC when filling the sample. The electric field distribution indicates that TMMSC can confine the sample in the microfluidic channel, enhancing the interaction between THz waves and the sample. Figure 8b shows the frequency shift relationship of cells in different tissues. It can be seen that the frequency shift of cancer cells is more significant than that of normal cells. In this case, the cancerous blood cells shifted to a lower frequency of 1.622 GHz and red-shifted by 348.4 GHz compared to the null sensor. In contrast, the normal blood cells were red-shifted by 337 GHz. Therefore, according to the shift of frequency, a mathematical model can be established for qualitative and quantitative analysis. This is of great practical importance for the commercial application of the sensor.

### 3.6. Manufacturing Engineering

**Figure 9:**
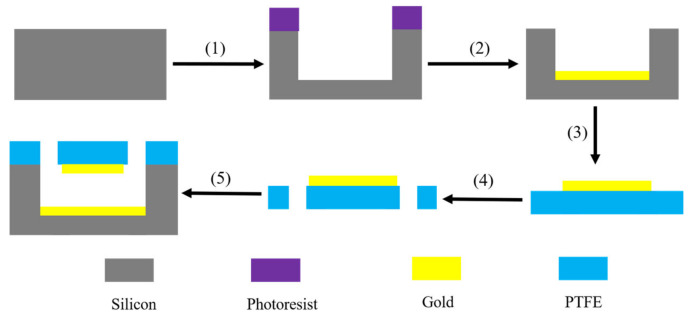
Process flow including (1) photolithography and etching to form microcirculation channels in the buffer layer; (2) production of metallic reflective layers; (3) creating a metal resonance pattern on a cover plate; (4) punching of holes in the cover plate; (5) bonding of the silicon substrate and the cover plate for encapsulation.

The terahertz microfluidic metamaterial sensor in this paper can be fabricated PDMS soft lithography technology. The process flow was shown in Figure 9. The specific steps include homogenization, exposure, development, silane treatment, glue filling, and bonding. First, we dropped the negative photoresist su8-2050 onto the silicon wafer and spun it. Then, we placed the mask on the silicon wafer and pressed it with glass to transfer it to the exposure table. Then, we separated the mask and the wafer, washed off the uncrosslinked photoresist, and cleared the wafer surface with a small amount of isopropanol. Next, we put the chip template in a vacuum dryer, added silane reagent to silane it, and completed the fabrication of the chip male mold. The PDMS prepolymer and initiator were evenly mixed and introduced into the die, and then the PDMS substrate was stripped for chip cutting and punching. The the cut PDMS substrate and glass slide were both put into the plasma cleaning machine for plasma cleaning treatment, and then stuck together tightly, and we used the oven to strengthen and bond them.

## 4. Discussion

In this study, a terahertz metamaterial microfluidic sensor for the detection of trace liquids is proposed based on microfluidic technology. The particle swarm optimization algorithm has accelerated the determination of the optimal structural parameters of the sensor. In the actual detection of the sensor, the polarization sensitivity and incidence angle sensitivity of the sensor affect the real detection characteristics. The M1 mode of the device is polarization-insensitive and incident angle-insensitive. This means that no matter which angle the terahertz wave is incident at, it will have little or no impact on the experimental results, thus ensuring the accuracy of the experimental results. In addition, the simulation results show that when the height of the microfluidic channel is 3 μM, the detection ability is the best. In this experiment, three kinds of cancer cells were simulated, and whether the cells were normal was judged by the different resonance offsets generated by the sensor chip using different cell detection methods. The device can direct the detected object into the microfluidic channel, simplifying the experimental process, and the experimental results have strong accuracy.

At present, the detection of terahertz metamaterial sensors is still in the basic research stage. There are detection examples of terahertz metamaterial sensors, most of which detect a single kind of mixture, but the detection of a substance in the mixed sample has not been considered. In this study, the frequency shift of the absorption curve generated by the detection of cancerous cells relative to the empty sensor is more than 330 GHz. At present, the laboratory is equipped with a terahertz time domain spectrometer with a resolution of 8 GHz, which can distinguish the detection substances of a single cell. From the frequency shift of the absorption curve, it can be seen that the offset between the microfluidic sensor and the mixture of MCF-7, HeLa and blood is less than 8 GHz, and particularly in the actual experimental process, the manufacturing error and experimental error and other factors will affect the experimental results, and it will be difficult to identify the mixture. However, we believe that with the gradual optimization of devices, the improvement of detection ability and the progress of manufacturing technology, some of the substances in the mixed sample will be identified and detected in the future.

At present, some research results have been used to manufacture microfluidic sensor chips through silicon wafer lithography process, PDSM soft lithography technology, and other manufacturing processes. In the actual manufacturing process, due to manufacturing errors, it is normal for the test results of the sensor chip to deviate from the simulation results. In the follow-up work, we will consider using PDSM soft lithography technology to manufacture the sensor chip. Combined with a terahertz time-domain spectrometer, the peak change and resonance shift of the device to different cell substances were observed.

## 5. Conclusions

In this paper, we presented a metamaterial-based microfluidic sensing chip for ultrasensitive detection of biomolecules at terahertz bands. The unit structure includes a PTFE cover layer, windmill model microstructure, micro flow channel, metallic reflective layer, and Si buffer layer. The sensor has three resonance peaks in the 1.5–3.0 THz range, with all the absorption peaks above 90%. The study was conducted to evaluate the physical mechanism, stability, and sensing performance of the proposed sensor. It was found that the first resonance peak (M1) is the best choice for sensing signals with Q-factor, sensitivity, and FOM values of 23,859 GHz/RIU, and 10, respectively. In addition, by filling the microfluidic tract with several types of cancer cells, TMMSC can correctly identify the cell types. The study in this paper will provide a reference for enriching the diversification of terahertz sensor devices and exploring non-destructive testing fields such as biomedicine, and the quality and safety of agricultural products.

## Figures and Tables

**Table 1:** Structural parameters of TMMSC.

Parameters	P	r	w	h1	h2	h3	t	d
Value (μm)	80	15	4	50	3	500	0.2	15

**Table 2:** Comparison of the proposed sensor performance parameters with other sensors reported in the literature.

Ref.	Resonance (THz)	Q-Factor	Sensitivity (GHz/RIU)	FOM
[36]	0.28	2.9	7.4	-
[37]	0.88	50.72	149.5	5.69
[38]	2.865	21.85	514.28	2.8
[39]	0.88	-	220.7	1.52
[40]	1.966	35	495	8.9
This work	1.97	23	859	10

**Table 3:** Refractive index of normal and cancer cells.

Cell Type	Refractive Index		Ref.
	Normal Cells	Cancer Cells	
Blood	1.376	1.39	[38]
Hela	1.368	1.392	[43]
MCF-7	1.387	1.401	[43]

## Data Availability

All data that support the findings of this study are included within the article.
